# Combining Experimental and Modeling Approaches to Understand Genotype x Sowing Date x Environment Interaction Effects on Emergence Rates and Grain Yield of Soybean

**DOI:** 10.3389/fpls.2020.558855

**Published:** 2020-09-02

**Authors:** Jay Ram Lamichhane, Jean-Noël Aubertot, Luc Champolivier, Philippe Debaeke, Pierre Maury

**Affiliations:** ^1^INRAE, Université Fédérale de Toulouse, Castanet-Tolosan, France; ^2^Terres Inovia, Institut Technique des Oléagineux, des Protéagineux et du Chanvre, Castanet-Tolosan, France; ^3^Université Fédérale de Toulouse, INRAE, INP-ENSAT Toulouse, Castanet-Tolosan, France

**Keywords:** crop emergence, genotype x environment interactions, *Glycine max*, non-emergence causes, seedbed conditions, seedling mortality

## Abstract

Soybean emergence and yield may be affected by many factors. A better understanding of the cultivar x sowing date x environment interactions could shed light into the competitiveness of soybean with other crops, notably, to help manage major biotic and abiotic factors that limit soybean production. We conducted a 2-year field experiments to measure emergence dynamics and final rates of three soybean cultivars from different maturity groups, with early and conventional sowing dates and across three locations. We also measured germination parameter values of the three soybean cultivars from different maturity groups under controlled experiments to parametrize the SIMPLE crop emergence model. This allowed us to assess the prediction quality of the model for emergence rates and to perform simulations. Final emergence rates under field conditions ranged from 62% to 92% and from 51% to 94% for early and conventional sowing, respectively. The model finely predicted emergence courses and final rates (root mean square error of prediction (RMSEP), efficiency (EF), and mean deviation (MD) ranging between 2% to 18%, 0.46% to 0.99%, and −10% to 15%, respectively) across a wide range of the sowing conditions tested. Differences in the final emergence rates were found, not only among cultivars but also among locations for the same cultivar, although no clear trend or consistent ranking was found in this regard. Modeling suggests that seedling mortality rates were dependent on the soil type with up to 35% and 14% of mortality in the silty loam soil, due to a soil surface crust and soil aggregates, respectively. Non-germination was the least important cause of seedling mortality in all soil types (up to 3% of emergence losses), while no seedling mortality due to drought was observed. The average grain yield ranged from 3.1 to 4.0 t ha^−1^, and it was significantly affected by the irrigation regime (*p* < 0.001) and year (*p* < 0.001) but not by locations, sowing date or cultivars. We conclude that early sowing is unlikely to affect soybean emergence in South-West of France and therefore may represent an important agronomic lever to escape summer drought that markedly limit soybean yield in this region.

## Introduction

Several socio-economic reasons are behind the need to increase soybean (*Glycine max* L.) acreage in the European Union. These reasons include the need to reduce import dependency of soybean for feed from the American continent, or to satisfy the increasing demand for locally produced, non-genetically modified protein crops (Bertheau and Davison, 2011). In addition, there are agronomic and environmental reasons that further trigger interests on leguminous crops in general, and soybean in particular. For example, soybean is able to fix atmospheric nitrogen with lower requirement of inorganic nitrogen input (Hungria and Mendes, 2015). Other advantages of growing soybean in the European Union could be a lower need for synthetic pesticides, including those used for seed treatments to control pests and diseases for two reasons: i) first pesticide seed treatment is incompatible with soil or seed inoculation of bacteria that promote nodulation (Campo et al., 2009; Zilli et al., 2009), and ii) soybean is still grown on a small surface in Europe, and in France it is often introduced in diversified cropping systems – especially rotations with maize and wheat—once every 5 to 6 years (Lecomte and Wagner, 2017). Indeed, synthetic pesticide input in France is lower for soybean, not only compared to many non-leguminous crops including maize, oilseed rape or cereals, but even compared to field pea, the most important leguminous crop grown in Europe (Agreste, 2019; Lamichhane et al., 2020b). A higher focus on soybean, at the expense of high-input crops, may thus help reduce the amount of synthetic inputs in agriculture and improve environmental sustainability.

Despite all benefits soybean may provide, the soybean acreage in France, and in the European Union in general, is almost non-existent compared to cereals (FAOSTAT, 2018). The lack of economic competitiveness (caused by a low average yield) of this crop compared with cereals has been reported as the key factor hindering its wide adoption in European cropping systems (Labalette et al., 2010). One of the ways to increase average yield of this crop is to reduce key biotic and abiotic factors that affect soybean yield through sowing date adaptations. For example, late sowings often limit soybean yield potential because the flowering and seed filling phase coincides with periods characterized by elevated summer temperatures and frequent drought events (Zhang et al., 2010; Hu, 2013; Clovis et al., 2015). This is especially true for southern European regions including South-West of France which is one of the two key French soybean production regions (Terres Inovia, 2019). Summer drought in these regions has been reported as the main limiting factor that affect the flowering and seed filling phase, critical for soybean yield (Maury et al., 2015). In contrast, an early sowing, as compared with conventional sowing dates, may provide higher crop yield in two ways across these regions (Schoving, 2020; Schoving et al., 2020): (i) by allowing soybean to produce more pods and seeds due to early flowering, at a less advanced vegetative stage and (ii) by allowing the crop to escape water deficit around flowering and seed filling phase. At the same time, the emergence quality of soybean can be affected due to shifting in sowing dates, especially for early sowings, which increase crop exposure to lower soil temperatures. Therefore, a better understanding of the effect of early sowings on soybean emergence is of paramount importance to ensure economic viability of this crop.

A number of previous studies showed that field crop emergence in France is mainly affected by seedbed conditions (temperature, humidity, and seedbed structure) rather than by cultivar characteristics (i.e., seed and seedling characteristics; Constantin et al., 2015; Dürr et al., 2016). However, these studies did not focus on soybean, and little is known about the potential effects of soybean cultivars on the quality of their establishment, especially under a wider range of pedo-climatic conditions and early sowing dates. The only study focusing on soybean crop establishment in South-Western France (Lamichhane et al., 2020a), based on a single sowing date (conventional one), using a single crop genotype (cv. ES Pallador) and in a single year (2018), showed that, for plots under conventional tillage, seedling mortality under mechanical obstacles (i.e. soil aggregates) were the most important stress factor causing emergence losses of soybean.

Crop models are important decision support tools to investigate the potential effects of cropping practices, including optimal sowing dates, on crop development and yield (Waha et al., 2012; Wolf et al., 2015; Dobor et al., 2016; Adnan et al., 2017). However, very few crop models have been developed and used to date for an in-depth understanding of the seed germination and seedling emergence process as affected by cropping practices and pedo-climatic conditions. More specifically to the crop emergence phase, the SIMPLE model (Dürr et al., 2001) has been reported as the most robust and comprehensive model to study dynamics of seed germination and seedling emergence in interaction with cropping practices (the choice of sowing date, crop genotype, sowing depth etc.) and seeded physical conditions (Constantin et al., 2015; Dürr et al., 2016; Lamichhane et al., 2019; Lamichhane et al., 2020a). Besides simulating the seedling emergence dynamics and final emergence rates, the SIMPLE model identifies the causes of non-emergence (Constantin et al., 2015; Dürr et al., 2016). This is very useful because identification of the causes of non-emergence under field conditions is not only time consuming and resource intensive but also a practically difficult task (Dürr et al., 2016). This is because seed germination and pre-seedling emergence phase occur under the soil surface, the investigation of which requires a very timely intervention in the field aiming at identifying the causes of non-emergence. A little delay in this intervention will result in the lack of seed or seedling part detection due to rotting, predation or other reasons that do not allow understanding of the non-emergence causes. Nevertheless, a timely intervention under field conditions is most often difficult as unfavorable meteorological conditions (e.g. rainfall, too cold or warm conditions) often limit field access (Dürr et al., 2016; Lamichhane et al., 2019). This difficulty is further exacerbated when field experiments are conducted across different sites requiring concomitant measurements. Coupling experimental and modeling approaches thus may allow to make a comprehensive study as the use of crop emergence models provides complementary data to field experiments.

The objectives of this study were to: i) perform laboratory experiments to generate germination parameters of three soybean cultivars from contrasted maturity groups, needed to parametrize the SIMPLE model; ii) conduct field experiments to measure emergence dynamics and final emergence rates of the same soybean cultivars across two sowing dates (i.e. early and conventional) and three different environments over a 2-year period; iii) analyze any potential correlation between the final rates of soybean emergence and grain yield; iv) evaluate the prediction quality of the SIMPLE model by using the independent dataset generated by field experiments; and v) determine genotype x sowing date x environment interaction effects on final emergence rates and identify key causes of non-emergence *via* simulations.

## Materials and Methods

### Overview of the SIMPLE Crop Emergence Model

A comprehensive description including the functioning of the SIMPLE model and the list of equations, parameter, and input and output variables has been previously provided (Dürr et al., 2001). In brief, the model predicts germination and emergence processes after sowing, as a function of seed and seedling characteristics, sowing depth distribution, and seedbed physical conditions (temperature, humidity, and structure). The model has previously been parameterized and positively evaluated for a number of crop species: wheat, sugar beet, flax, mustard, French bean, oilseed rape (Dürr et al., 2001; Dorsainvil et al., 2005; Moreau-Valancogne et al., 2008; Dürr et al., 2016), soybean (Lamichhane et al., 2020a), several catch crops (Constantin et al., 2015) and a plant model *Medicago truncatula* (Brunel et al., 2009). Here, we mainly focus on the model’s key features and the input variables measured for soybean crop, without presenting the already published equations.

SIMPLE creates 3D representations of seedbeds with sowing depth distribution and the size, number, and position of soil aggregates as input variables. Soil temperature at the mean sowing depth and daily soil water potential in 0 to 3, 3 to 5, and 5 to 10 cm layers are also used as input variables for simulations, along with plant characteristics for germination and seedling growth. Seeds are placed into virtual numerical seedbeds at random using a sowing depth distribution provided by the user. The model predicts germination and emergence, seed by seed, at a daily time step. The time required for germination of the seed *i* is chosen at random in a distribution of thermal times needed to reach germination for the considered seed lot. Hydrothermal time since sowing is calculated using a base temperature for germination (Tb), provided that the soil water content at the seed sowing depth is above a base water potential (Ψb). The Tb and Ψb constants for germination are input variables. If seed i has not germinated after a given time (often fixed at 30 days), the model considers that it will never germinate. If the seed germinates, then a seedling grows from the seed. To better include the effect of early water stress on seedling growth, we added a water stress function to the SIMPLE model, which reduces emergence after germination (Constantin et al., 2015). With this function, the fate of seedlings is determined by considering soil water potential in the soil layer in which the radicle grows in the two days following germination. If it is lower than Ψb, the seedling does not emerge and dies the following day. If this is not the case, the time it takes for the seedling to reach the soil surface after germination is calculated by SIMPLE based on its seed’s sowing depth, the length of the pathway the shoot takes through the aggregates, and the shoot’s elongation function, whose parameters are input variables. The probability of the seedlings remaining blocked under aggregates depends on the size and position of the soil aggregates in the seedbed, i.e. lying on the surface or below it. Soil surface crusting depends on cumulative rainfall after sowing; a proportion of seedlings remain blocked under the crust depending on daily crust water content (dry or wet soil surface). Simulations were run for 1000 seeds to predict the emergence rate over time, including final emergence rates. The causes of non-emergence simulated by SIMPLE are (i) non-germination, (ii) death of seedlings caused by water stress after germination and (iii) mechanical obstacles (soil aggregates or a soil crust). The SIMPLE model does not consider the impact of: i) high temperatures, ii) the excess in soil moisture, and iii) biotic stresses, such as soil-borne pests and diseases, all of which could affect seed germination and seedling emergence.

### Laboratory Experiments

Because the SIMPLE model has been already parameterized for soybean (Lamichhane et al., 2020a), we used parameters related to early seedling growth from the database associated with the SIMPLE model. This is because parameters related to seed germination and heterotrophic growth most often differ significantly only at the species level and not much at the genotype level (Tribouillois et al., 2016). Consequently, we used from our database (Lamichhane et al., 2020a) the parameter values related to hypocotyl and radicle elongation, and seedling mortality rates under soil aggregates for the cultivars used in this study. In contrast, we estimated parameters of the model related to seed germination at different temperatures and water potentials of all three soybean cultivars used in this study that have contrasting characteristics (Schoving et al., 2020): cv. Ecudor belongs to the maturity group II (MGII), is characterized by indeterminate growth, and has big round leaves; cv. Isidor belongs to the MGI, is characterized by semi-determinate growth, and has big round leaves; and cv. Santana belongs to the MG I/II (in between), is characterized by indeterminate growth and has rounded oval leaves. These MGs are mostly grown in South-West of France, which is the main French soybean production basin. The procedure used for soybean germination has been published (Lamichhane et al., 2020a). Briefly, four replicates of 25 seeds, for each temperature, were put in 90 mm Petri dishes on Whatman^®^ filter paper of the same size placed both below and above the seeds and imbibed with 8 ml deionized water. The dishes were incubated at 3°C, 6.5°C, 10°C, 15°C, 20°C, 25°C, 30°C, 35°C, 37.5°C, 40°C, and 43°C and temperatures were recorded hourly with sensors. Depending on the temperature of incubation, seed germination was assessed up to three times per day until no further germination was observed. A given seed was considered as germinated when the radicle was >3 mm. After each observation, the germinated seeds were removed from the dishes. The base temperature for germination was determined by fitting a Gompertz function to the observed germination rates as described previously (Brunel et al., 2009). Adjustments of germination dynamics were made for each temperature and values of Tb for germination were defined as the X-intercept of the linear regression between the temperature and germination rate (Gummerson, 1986; Dahal and Bradford, 1994). We determined the range of temperatures for which a strong linear relationship existed between germination rates (1/time to reach 20, 40, 50, 60, 80, and 90% germination) to calculate the X-intercepts. Base temperature was defined as the intercept with the X-axis that corresponds to the temperature fitted value at which no germination occurs. The optimum temperature for germination, corresponding to the maximum germination speed, was determined using a non-linear equation (Yin et al., 1995), where maximum and minimum temperatures were found as function parameters.

Seed germination response to different water potentials (0,−0.10,−0.25, and −0.50 MPa) was tested at 20°C by slightly modifying the previously described method (Lamichhane et al., 2020a). However, we did not test water potentials below −0.50 MPa as no soybean seed germination occur below this threshold (Lamichhane et al., 2020a). The same number of seeds per replicate as described above was used. The seeds were disinfected by using 2.6% sodium hypochlorite solution for 10 min followed by two rinses in distilled water. Twenty-five disinfected seeds were laid onto flat Whatman filter paper in 90 mm Petri dishes with 15 mL of osmotic solutions of high molecular weight PEG (Polyethylene glycol 8000, ref. Fisher Scientific) to control water potential (Michel, 1983). Only 8 ml of deionized water used for control treatments. The base water potential for germination (*Ψ_b,germ_*) was determined by fitting a Gompertz function to the observed germination rates and values of *Ψ_b,germ_* were defined as the X-intercept of the linear regression between the temperature and germination rate as described above.

### Field Experiments

#### Description of Experimental Site, Seedbed Preparation, and Sowing Operations

The experiments were carried out in 2013 and 2014 across three experimental sites: En Crambade (43.25°N, 1.39°E), an experimental station of Terres Inovia located South East of Toulouse; Mondonville (43.40°N, 1.17°E), an experimental station of Euralis Semences located North-West of Toulouse; and Rivières (43.54°N, 1.59°E), an experimental station of RAGT 2n, located North-West of Toulouse. These experimental sites were chosen because of their contrasting pedo-climatic conditions that could affect the quality of soybean establishment (**Table 1**). Overall, five soil types, as defined by FAO (2006), were present across the study sites: clay in En Crambade for both sowings in 2013; silty loam in En Crambade for the early sowing in 2014, and in Mondonville for all sowings in 2013 and 2014; silty clay loam for early 2013 sowing and for both 2014 sowings in Rivieres; and clay loam for conventional sowings in Rivières in 2013; and silty clay in En Crambade for the conventional sowing in 2014.

**Table 1:** Physico-chemical properties of the observed seedbeds for the three locations, 2 years and two sowing dates considered in the study.

Seedbed/sowing characteristics	En Crambade				Mondonville				Rivières			
	2013		2014		2013		2014		2013		2014	
	ES (clay soil)*	CS (clay soil)*	ES (silty loam)*	CS (silty clay)*	ES (silty loam)*	CS (silty loam)*	ES (silty loam)*	CS (silty loam)*	ES (silty clay loam)*	CS (clay loam)*	ES (silty clay loam)*	CS (silty clay loam)*
**Soil granulometry**												
Clay (g·g^−1^)	0.6	0.6	0.25	0.41	0.15	0.15	NT	0.15	0.34	0.30	0.36	0.36
Silt (g·g^−1^)	0.37	0.37	0.51	0.47	0.64	0.68	NT	0.68	0.45	0.48	0.43	0.43
Sand (g·g^−1^)	0.03	0.03	0.24	0.12	0.17	0.15	NT	0.15	0.18	0.21	0.18	0.18
**Soil chemical characteristics**												
Total organic Carbon (g·g^−1^)	0.0131	0.0131	0.0085	0.0112	0.0062	0.0063	NT	0.0063	0.0088	0.0085	0.008	0.008
Total nitrogen (g·g^−1^)	0.0018	0.0018	0.0011	0.0014	0.0009	0.0008	NT	0.0008	0.001	0.001	0.0011	0.0011
C/N ratio	7.28	7.28	7.73	8.00	6.89	7.88	NT	7.88	8.80	8.50	7.27	7.27
pH	6.6	6.6	8.6	8.4	6.5	6.8	NT	6.8	6.8	6.7	7.2	7.2
Organic matter (g·g^−1^)	0.022	0.022	0.022	0.022	0.011	0.011	NT	0.011	0.015	0.015	0.014	0.014

ES, early sowing; CS, conventional sowing; NT, not tested.

*Soil classification as defined by FAO (2006).

After the harvest of the preceding crop (wheat in En Crambade and Mondonville in both years, maize and straw cereals in Rivières in 2013 and 2014, respectively), conventional tillage was performed with 4-body plough at 25 cm depth, followed by a passage of Vibrashanks at 7 to 10 cm depth, and that of Flat harrow at 5 to 7 cm depth. Soybean cultivars (Ecudor, Isidor, and Santana) were sown in 64 m^2^ blocks (each of 10 m long and 6.4 m wide, 3 blocks in total/cultivar) and at 3 cm depth with 45 seeds m^−2^ and with 50 cm inter-row distance. The experimental designs used were completely randomized blocks. For each year, an early and control (hereafter referred to as conventional sowing) sowing dates were compared. In 2013, early sowings were performed on 15^th^ March, 22^nd^ March, and 22^nd^ March while conventional sowings were made on 25^th^ April, 27^th^ May, and 6^th^ May, in En Crambade, Mondonville, and Rivières, respectively. In 2014, early sowings were performed on 14^th^ March in En Crambade and 18^th^ March in Rivières (there was no early sowing in 2014 in Mondonville) while conventional sowings were made on 30^th^ April, 6^th^ May, and 6^th^ May, in En Crambade, Mondonville, and Rivières, respectively. The seed lot of a given genotype was the same across locations for a given year, while it was different between years. Soil samples from the 0- to 30-cm soil horizon were taken immediately after sowing and sent to a laboratory to perform physico-chemical characterization (**Table 1**). Because seedling damages due to vertebrate pests (birds and wild rabbit in particular) are a key problem across south-West France, the experimental plots were covered with a protective nylon net, placed at approximately 30 cm above the ground level which did not affect rain input to the soil or soil temperature.

#### Determination of Seedling Emergence Rates

Seedling emergence was counted in 3 m^2^/block (2 linear meters/row, 3 rows in diagonal, for a total of 9 m^2^/plot) delimited with plastic pegs. A seedling was considered emerged when cotyledons were clearly visible over the soil surface (i.e. VE stage; Fehr and Caviness, 1977). Although we aimed at determining the final emergence rates, we made different field counting to determine the kinetic of seedling emergence whenever possible depending on time availability and weather conditions that often limit field access.

#### Grain Yield Determination

For each sowing date, the experimental plots were managed under two irrigation regimes following soybean emergence: plots with and without irrigation. Irrigated plots were optimally irrigated using a decision tool (Terres Inovia, 2019), resulting in water applications from 65 to 161 mm during the reproductive stage (from R1: first flower to R7: beginning of maturity), critical period for soybean in terms of water need (Montoya et al., 2017). Finally, following the completion of the crop maturity, the crop was harvested. The area harvested ranged from 11 to 21 m² depending on the study site (average 16 m²). To remove any edge effects, we excluded the two external rows as well as ~0.5 m of two extremities of the plot by removing the plants before harvest. The soybean grains were dried up to remove the residual moisture and grain yield was determined for each sowing date across all locations. Because the yield is usually expressed at 13% grain humidity for soybean, grain weights were corrected to 130 g·kg^−1^ moisture content.

### *In Silico* Experiments

#### Model Evaluation

The emergence rates (along the emergence courses, including the final rate) observed in the field experiments were used to assess the prediction quality of the model. The seedbed structure and the sowing depth adopted were the same as those commonly obtained by farmers in the region as previously published (Lamichhane et al., 2020a). Based on this information, we chose a seedbed structure and a sowing depth variability, similar to the one already described in our database associated with the SIMPLE model. This database describes different types of seedbeds and sowing depths. However, all other field data needed as input variables of the model, including seedbed weather conditions, were directly collected. To this objective, soil temperatures over the sowing depth were recorded using climate sensors (Thermo 2000, Canada) from sowing until the completion of emergence. These sensors delivered temperatures every three hours. Rainfall data were obtained from an automatic meteorological station located at or near the experimental site. As for the potential presence or absence of water stress in the seedbed at sowing, we used two indicators: the water content of the seedbed at sowing depth – determined by the difference between humid and dry masses of the soil (in En Crambade); the quantity of cumulative rainfall 7 days before sowing (in Mondonville and Rivières). Given that the rate of water evaporation is still low across the study sites for the sowing dates considered, and given the prevalence of clay or loamy soil across the study sites, we supposed that there was no water stress in the seedbed at and following sowing, if at least 10 mm cumulative weekly rainfall occurred. The cumulative degree-days (°Cd) were calculated from sowing (time 0) as the sum of the average daily air temperature minus germination base temperature of soybean times one day. Simulations were then performed and the results were compared with the emergence rates observed in the field.

Three statistical criteria – model efficiency (EF), root mean square error of prediction (RMSEP) and mean deviation (MD) – were calculated to assess the quality of model predictions for germination and emergence.

(1) $$EF=1-\frac{\sum_{j=1}^{n}{\left(Pj-Oj\right)}^{2}}{\sum_{j=1}^{n}{\left(Oj-\bar{O}\right)}^{2}}$$

(2) $$RMSEP=\sqrt{\sum_{j=1}^{n}[(Pj-Oj}{)}^{2}/n]$$

(3) $$MD=\frac{1}{n}\sum_{j=1}^{n}\left(Pj-Oj\right)$$

where EF (ranges from −∞ to 1) represents model efficiency relative to the mean of observed data and is = 1 for a perfect model prediction. The more EF approaches 1, the more is the match between observed and predicted values. Pj and Oj are predicted and observed values, respectively, n is the number of observations, and Ō is the mean of observed values. RMSEP is the square root of mean squared error. The unit of this criteria is the same as that of the analyzed variables. MD provides model deviation that is a measure of the tendency of the model to under- or overestimate predicted values compared with observations. A negative value indicates that the majority of predicted values are less than the observed ones (i.e. predicted time courses are ahead of the observed ones).

#### Simulation Studies

We performed 10 simulations/cultivar for each sowing date/location/year. Each simulation contained one seedbed with 1000 seeds/cultivar for each sowing date/location/year (1,000 × 360 = 360 000 simulations in total). Because SIMPLE is a stochastic model, the outcomes of these simulations for each cultivar were variable and this variability were considered for further statistical analyses. This was needed to investigate the potential effects of soybean cultivars, sowing dates and locations on final emergence rates, and to determine causes of non-emergence.

### Statistical Analyses

Statistical analyses were performed to test the potential effects of different treatments (cultivars, sowing dates, locations, years, irrigation regimes) on final emergence rates, causes of non-emergence, and final grain yield. One-way ANOVA followed by a Tukey’s HSD *post-hoc* test was performed to assess significant differences between treatments. A two-way ANOVA was applied to determine any possible interaction effects among treatments on the tested variables. All statistical analyses were applied using the R software (Hothorn and Everitt, 2009). As described previously (Lamichhane et al., 2020a), the quality of field emergence was analyzed by establishing three classes: poor (<50%), good (50–75%), and very good (>75%) and the frequency (%) of each class was calculated.

## Results

### Laboratory Experiments

**Figure 1:**
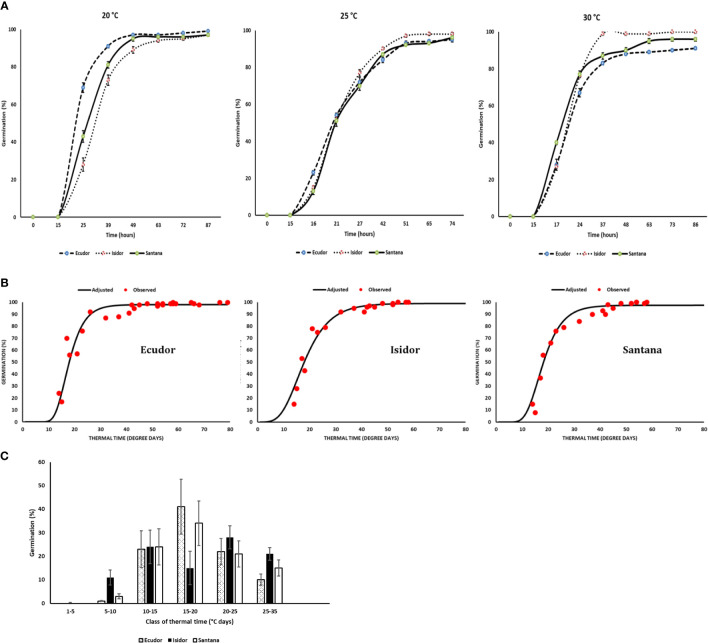
Germination measurements of three soybean cultivars in the laboratory (n = 4). Seed germination at 20°C, 25°C, and 30°C (Vertical bars reported in the figure represent standard deviations; **(A)**; combined values of seed germination dynamics observed at 20°C, 25°C, and 30°C in relation to thermal time **(B)**; distribution of thermal times for seed germination **(C)**. The base temperature for germination was 4°C, 3°C, and 3.5°C for cvs. Ecudor, Isidor, and Santana, respectively.

The raw data on the response of the three cultivars to the tested temperatures and water potentials are presented in **Supplementary Material S1**. Germination rates and time-courses were different in relation to temperature among the three cultivars tested, although the ranking was not consistent across incubation temperatures (**Figure 1A**). For example, cv. Ecudor germinated faster at 20°C, followed by cvs. Santana and Isidor, while this ranking was reversed at 30°C with cv. Isidor germinating better followed by cvs. Santana and Ecudor. The average final germination rate was slightly higher for cv. Isidor (99%), while it was slightly lower for cvs. Ecudor and Santana (97% each). The optimum temperature for germination was 28°C, 30°C, and 30°C for cv. Ecudor, Isidor and Santana, respectively (data not shown). The calculated base temperature for seed germination was 4°C, 3°C, and 3.5°C for cvs. Ecudor, Isidor and Santana, respectively. The germination curve, expressed as a function of thermal time, well-grouped the germination results obtained at 15°C, 20°C, and 25°C for cv. Ecudor and 15°C, 20°C, 25°C, and 30°C for cvs. Isidor and Santana to which a Gompertz function was fitted (**Figure 1B**). No significant differences were observed among the cultivars, in terms of thermal time to reach 50% germination, which was 18°Cd for all three cultivars and that almost all seeds germinated very rapidly before 40°Cd (**Figure 1B**). The resulting distribution of thermal times required for germination, calculated from the cultivar germination curves and base temperature, showed that all three cultivars germinate at each class of thermal time, although to a different extent (**Figure 1C**).

**Figure 2:**
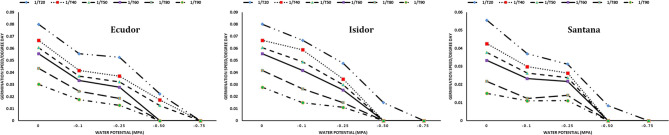
Seed germination speed of soybean genotypes at different water potentials. 1/T20, 1/T40, 1/T50, 1/T60, 1/T80, and 1/T90 indicate germination speed to reach 20%, 40%, 50%, 60%, 80%, and 90% germination, respectively.

When PEG 8000 was used, the rates of seed germination were the following: 94%, 94% and 85% at −0.10 MPa; 88%, 81%, and 88% at −0.25 MPa; and 51%, 23% and 19% at −0.50 MPa for cultivar Ecudor, Isidor, and Santana, respectively. The germination speed decreased with increasing water potential. The base water potential of the genotype ranged from −0.75 MPa for the fastest germinating seeds to −0.50 MPa for the slowest germinating seeds with an average value corresponding to −0.58, −0.56, and −0.71 MPa for cultivar Ecudor, Isidor, and Santana, respectively (**Figure 2**).

### Field Experiments

#### Soil and Weather Data

Results related to the average seedbed weather conditions are reported in **Table 2** while detailed weather conditions are presented in **Supplementary Material S2**. The average cumulated rainfall 30 days after sowing ranged from a minimum of 34 mm (En Crambade, in 2014) to a maximum of 125 mm (Mondonville, in 2013). Cumulated rainfall significantly differed (*p*<0.01) among years, but not between sowing dates with higher values in 2013 than in 2014 for both sowings. In contrast, no significant effects of locations, sowing dates or location x sowing date, location x year, sowing date x year or location x sowing date x year interactions were observed on average cumulated rainfall 30 days after sowing. The average sowing depth temperature 30 days after sowing ranged from a minimum of 11°C (early sowing date in En Crambade, in 2013) to a maximum of 17°C (conventional sowing date in En Crambade, in 2014). The average sowing depth temperature was the lowest in Rivières except for early sowings in 2013, while it was similar for other two locations except for conventional sowings in 2014. As expected, the average sowing depth temperature 30 days after sowing was higher for the conventional sowing date across all locations, and for both years. Indeed, there was statistically significant effect of locations (*p*<0.01), sowing dates (*p*<0.001), years (*p*<0.001), as well as location x sowing date (*p*<0.001), location x year (*p*<0.01) interactions. In contrast, no significant effect of sowing date x year interaction nor that of location x sowing date x year interaction (*p*>0.05) was observed on the average sowing depth temperature 30 days after sowing.

**Table 2:** Weather variables (mean values ± standard deviation; n = 30) of the study locations across different years and sowing dates.

Weather parameters	Location	2013		2014	
		ES	CS	ES	CS
Cumulated rainfall (mm) 30 das	En Crambade	88^c^ ± 3	101^a^ ± 4	64 ± 3	34^a^ ± 2
	Mondonville	66^a^ ± 2	125^c^ ± 5	NT	46^b^ ± 2
	Rivières	81^b^ ± 3	112^b^ ± 4	48 ± 2	77^c^ ± 4
**Overall significance level**					
Location	NS				
Sowing date	NS				
Year	**				
Location x sowing date	NS				
Location x year	NS				
Sowing date x year	NS				
Location x sowing date x year	NS				
**Weather variables**	**Location**	**2013**		**2014**	
		**ES**	**CS**	**ES**	**CS**
Temperature (°C) 30 days at the average sowing depth	En Crambade	11^a^ ± 3	16^b^ ± 2	13 ± 2	17^b^ ± 1
	Mondonville	12^a^ ± 3	16^b^ ± 2	NT	15^a^ ± 2
	Rivières	12^a^ ± 3	13^a^ ± 2	12 ± 2	15a ± 2
**Overall significance level**					
Location	**				
Sowing date	***				
Year	***				
Location x sowing date	***				
Location x year	**				
Sowing date x year	NS				
Location x sowing date x year	NS				

Means followed by the same letter are not significantly different at p < 0.05; ***p < 0.001; **p < 0.01.

ES, early sowing; CS, conventional sowing; NT, not tested; NS, not significant; das, days after sowing.

The threshold of cumulated weekly rainfall <10 mm triggering the water stress in the seedbed was observed only in four out of 11 cases: 5.4 and 9.0 mm for early and conventional sowing in En Crambade in 2013; 0.2 and 0.8 mm for early sowing in En Crambade and in Rivières, respectively, in 2014 (data not shown). Overall, the frequency of weekly cumulative rainfall <10 mm was very low for the sowings in 2013, while this frequency was higher for the sowings in 2014 for both sowing dates and across all locations (data not shown).

#### Seedling Emergence

**Figure 3:**
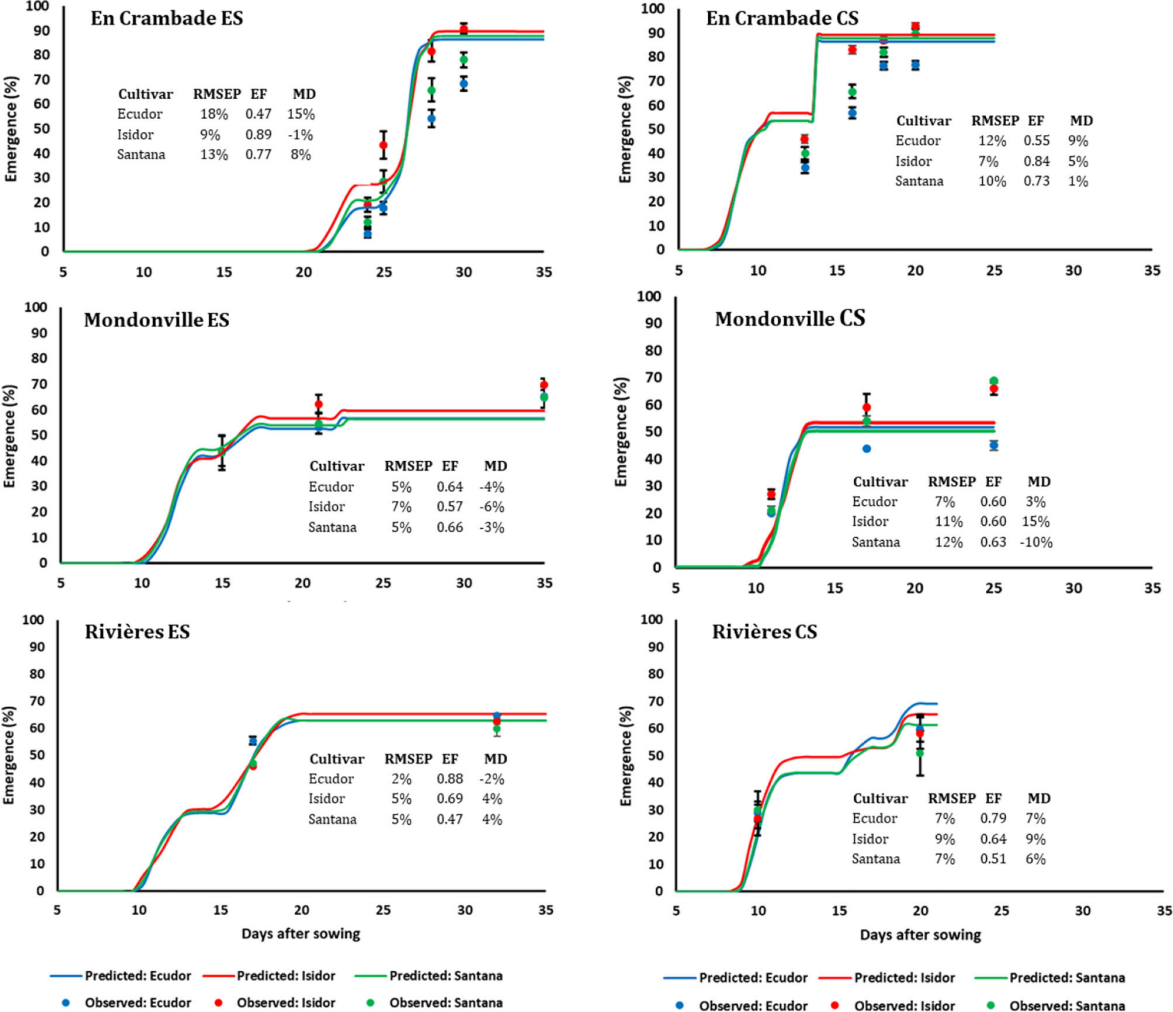
Simulated and observed emergence rates of three soybean cultivars for early (ES) and conventional (CS) sowing dates across three locations, in 2013. Vertical bars reported in the figure represent standard deviations (n = 9). EF, model efficiency; RMSEP, root mean square error of prediction; MD, mean deviation.

**Figure 4:**
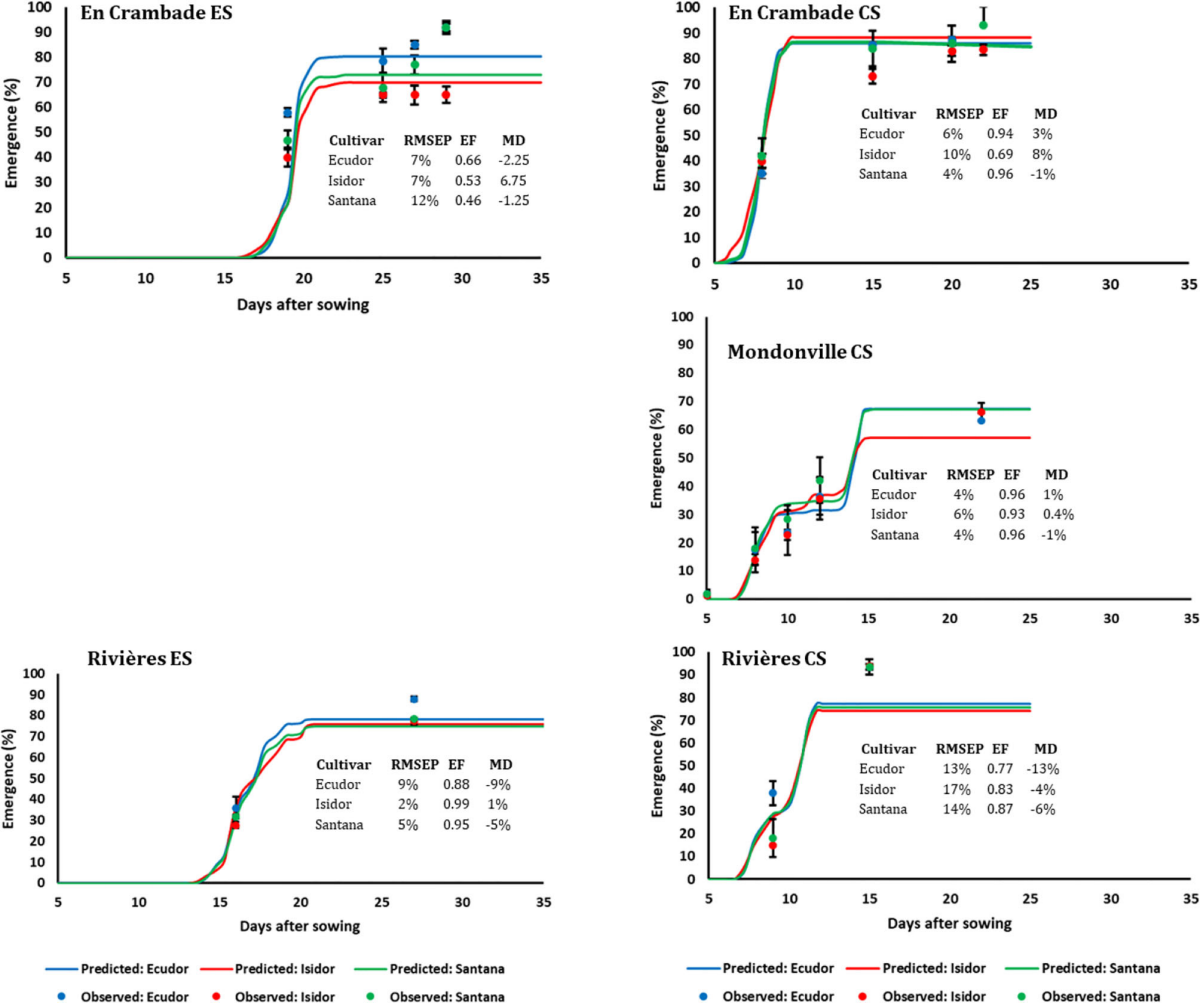
Simulated and observed emergence rates of three soybean cultivars for early (ES) and conventional (CS) sowing dates across three locations, in 2014. Vertical bars reported in the figure represent standard deviations (n = 9). EF, model efficiency; RMSEP, root mean square error of prediction; MD, mean deviation.

Seedling emergence dynamics under field conditions in 2013 and 2014 are reported in **Figures 3** and **4**, respectively. The final emergence rates ranged from 45% (for cv. Ecudor for conventional sowing in Mondonville, in 2013) to 93% (for cv. Isidor for conventional sowing in En Crambade, in 2013). Overall, the final emergence rate was the highest for Isidor, followed by Santana while it was the lowest for Ecudor, with some exceptions (i.e. both sowings in Rivières in 2013 and 2014, and early sowing in En Crambade, in 2014). For the sowings in 2013, the final emergence rates for the tested cultivars were generally higher in En Crambade, followed by Mondonville, while they were the lowest for Rivières. In contrast, the ranking, in terms of the final emergence rates, differed for the sowings in 2014, with En Crambade and Rivières having similar values and Mondonville having the lowest values. Overall, final emergence rates of a given cultivar differed between early and conventional sowings although this difference was small in some cases. Number of days from emergence to maturity of the three soybean cultivars grown over space and time under two irrigation regimes are reported in **Supplementary Material S3**.

#### Soybean Grain Yield

The average grain yield values are reported in **Table 3**. As expected, the average grain yield under irrigated condition was significantly higher (*p*<0.001) compared with non-irrigated plots (4.48 t ha^−1^ of irrigated vs. 3.56 t ha^−1^ of non-irrigated). Likewise, the average grain yield in 2014 was significantly higher (*p*<0.001) compared with that in 2013 (4.60 t ha^−1^ vs 3.56 t ha^−1^). Nevertheless, unlike for average seedling emergence rate (**Table 4**), no statistically significant effect of sowing dates, locations or cultivars was found on the average grain yield. This means that there was no positive correlation between the seedling emergence rate and the average grain yield of soybean across any locations and/or sowing dates.

**Table 3:** Observed mean grain yield of the three soybean cultivars used in this study across different locations and sowing dates under two irrigation regimes.

Effect	Variables	n	Average grain yield (t ha^−1^) ± SD	Significance level
**Irrigation regime**	Non irrigated	33	3.56 ± 0.80	***
	Irrigated	33	4.48 ± 0. 69	
**Locations**	En Crambade	24	4.25 ± 0.57	NS
	Mondonville	18	4.02 ± 1.03	
	Rivières	24	3.91 ± 1.03	
**Sowing date**	Early	30	3.91 ± 0.92	NS
	Conventional	36	4.14 ± 0.80	
**Cultivars**	Ecudor	22	4.14 ± 0.80	NS
	Isidor	22	3.91± 0.92	
	Santana	22	4.14 ± 1.03	
**Year**	2013	33	3.56 ± 0.80	***
	2014	33	4.60 ± 0.57	

***p < 0.001.

Grain yield is referred to yield with 13% of humidity.

NS, not significant

**Table 4:** Simulated mean final emergence rates (% ± standard deviation; n = 10) of the three soybean cultivars used in this study across different locations and sowing dates.

Location	Cultivar	2013		2014	
		ES	CS	ES	CS
En Crambade	Ecudor	86^a^ ± 0.52	87^ab^ ± 0.28	79^c^ ± 1	63^c^ ± 2
	Isidor	87^a^ ± 1	88^b^ ± 0.58	71^a^ ± 0.90	52^a^ ± 1.34
	Santana	86^a^ ± 1	86^a^ ± 0.83	75^b^ ± 0.76	59^b^ ± 1.13
Mondonville	Ecudor	49^a^ ± 1.61	84^ab^ ± 0.46	NT	65^b^ ± 0.89
	Isidor	51^a^ ± 1.55	85^b^ ± 0.65	NT	57^a^ ± 0.75
	Santana	51^a^ ± 0.6	83^a^ ± 0.85	NT	64^b^ ± 0.74
Rivières	Ecudor	62^a^ ± 1.12	81^a^ ± 1	79^a^ ± 1	66^b^ ± 1
	Isidor	63^a^ ± 0.85	81^a^ ± 1.32	78^a^ ± 1	58^a^ ± 1.56
	Santana	63^a^ ± 1	81^a^ ± 1.33	78^a^ ± 1.41	64^b^ ± 2
**Overall significance level**				
Location	***				
Sowing date	***				
Cultivar	***				
Year	***				
Location x cultivar	*				
Sowing date x cultivar	**				
Cultivar x year	***				

Means followed by the same letter are not significantly different within locations, cultivars, year or sowing dates at p< 0.05; ***p < 0.001; **p < 0.01; *p < 0.05.

ES, early sowing; CS, conventional sowing; NT, not tested.

### In Silico Study

#### Model Evaluation

The comparisons between predicted and observed emergence rates of the three soybean cultivars for early and conventional sowings in 2013 and 2014 are reported in **Figures 3** and **4**, respectively. Overall, the model finely predicted emergence rates, over emergence courses as well as final emergence rates. However, the predictive quality of the model differed not only between years and sowing dates and locations but also between cultivars within a given sowing date/location/year. Overall, the model efficiency ranged from 0.46 (cv. Santana for early sowing in En Crambade, in 2014) to 0.99 (cv. Isidor for early sowing in Rivières, in 2014).

#### Simulation Results

Simulated final emergence rates are reported in **Table 4** that further confirmed results of the field observations. We found statistically significant effects of location (*p*<0.001), sowing date (*p*<0.001), cultivar (*p*<0.001), and year (*p*<0.001) on final emergence rates of soybean cultivars used in this study. There was also a significant interaction effect of location x cultivar (*p*<0.05), sowing date x cultivar (*p*<0.01), and cultivar x year (*p*<0.001) on final emergence rates of soybean cultivars.

Results regarding the frequency of cases with <50%, 50% to 75%, and >75% emergence rates are presented in **Table 5**. Overall, the frequency of cases with <50% emergence ranged from 0–11%, 0–6%, and 0–5%, when analyzed by location, sowing date and year, respectively. Time to reach the maximum emergence was the highest for Mondonville followed by En Crambade while it was the lowest for Rivières (**Figures 3** and **4**). Time to reach the maximum emergence ranged from 27 (Rivières in 2014) to 35 days (Mondonville in 2013) for the early sowing and from 15 (Rivières in 2014) to 25 (Mondonville 2013) days for the conventional sowing depending on the seedbed weather conditions (data not shown).

**Table 5:** Emergence frequencies per classes of emergence rates when simulated by location, sowing date and year.

Variable	Location	Frequency (%) of emergence rate		
		<50	50–75	>75
Location	Crambade	0	17	83
	Mondonville	11	89	0
	Rivieres	0	50	50
Sowing date	Early	0	53	47
	Conventional	6	44	50
Year	2013	5	67	28
	2014	0	27	73

Results concerning major causes of non-emergence are reported in **Table 6**. Key causes of non-emergence differed across the locations and among sowing dates or between years. Seedling mortality due to a soil surface crust was the most important cause of non-emergence in Mondonville and Rivières for early sowings in 2013, followed by that due to soil aggregates and non-germination. In contrast, seedling mortality due to soil aggregates was the main cause of non-emergence in most cases, and was the least variable across all locations, followed either by a soil surface crust or non-germination, depending on locations. We did not find any seedling mortality due to drought as the cumulated rainfall from sowing to emergence was elevated (**Table 2**) with a relatively high rainfall frequency during this period **(****Supplementary Material S2****)**.

**Table 6:** Non-emergence causes (% ± standard deviation) based on the results of the simulations.

Causes of non-emergence	Location	2013						2014					
		ES			CS			ES			CS		
		Ecudor	Isidor	Santana	Ecudor	Isidor	Santana	Ecudor	Isidor	Santana	Ecudor	Isidor	Santana
Non-germination	En Crambade	3^a^ ± 0.37	1^a^ ± 0.32	3^b^ ± 0.33	3^b^ ± 0.38	1^a^ ± 0.21	3^b^ ± 0.19	3^b^ ± 0.12	1^a^ ± 0.16	3^b^ ± 0.51	3^b^ ± 0.74	1^a^ ± 0.17	3^b^ ± 0.6
	Mondonville	3^b^ ± 0.32	0.8^a^ ± 0.16	3^b^ ± 0.28	3^b^ ± 0.35	1^a^ ± 0.10	3^b^ ± 0.30	NT	NT	NT	3^b^ ± 0.39	0.84^a^ ± 0.19	3^b^ ± 0.72
	Rivières	3^b^ ± 0.55	1^a ±^0.08	3^b^ ± 0.15	3^b^ ± 0.76	1^a^ ± 0.24	3^b^ ± 0.46	3^b^ ± 0.49	0.82^a^ ± 0.15	3^b^ ± 0.40	3^b^ ± 0.18	1^a^ ± 0.21	3^b^ ± 0.28
	**Overall significance level**												
	Location	Sowing date	Cultivar	Year	Location x Cultivar	Sowing date x cultivar	Cultivar x year						
	NS	NS	***	NS	NS	NS	NS						
Soil aggregates	En Crambade	11^a^ ± 0.50	11^a^ ± 0.65	11^a^ ± 0.75	10^a^ ± 0.46	11^a^ ± 0.52	11^a^ ± 1	11^a^ ± 0.75	10^a^ ± 0.72	10^a^ ± 0.34	11^a^ ± 0.62	10^a^ ± 0.52	10^a^ ± 0.6
	Mondonville	14^b^ ± 0.78	13^ab^ ± 0.63	12^a^ ± 0.61	13^a^ ± 0.79	14^a^ ± 0.6	14^a^ ± 0.68	NT	NT	NT	13^a^ ± 1.20	14^a^ ± 0.73	13^a^ ± 0.60
	Rivières	13^a^ ± 0.4	14^a^ ± 0.91	13^a^ ± 0.80	13^a^ ± 0.48	13^a^ ± 0.84	12^a^ ± 1.15	13^a^ ± 0.44	13^a^ ± 1	13^a^ ± 1.36	13^a^ ± 0.26	13^a^ ± 1	13^a^ ± 1.4
	**Overall significance level**												
	Location	Sowing date	Cultivar	Year	Location x Cultivar	Sowing date x cultivar	Cultivar x year						
	***	NS	NS	NS	NS	NS	NS						
Crust	En Crambade	0 ± 0	0 ± 0	0 ± 0	0 ± 0	0 ± 0	0 ± 0	3^a^ ± 0.2	7^c^ ± 0.68	5^b^ ± 0.36	0 ± 0	0 ± 0	0 ± 0
	Mondonville	34^a^ ± 1.40	35^a^ ± 1.96	34^a^ ± 0.6	0 ± 0	0 ± 0	0 ± 0	NT	NT	NT	7^a^ ± 1	12^b^ ± 0.54	8^a^ ± 1
	Rivières	21^a^ ± 1.25	22^a^ ± 0.77	21^a^ ± 0.51	2^a^ ± 0.27	5^c^ ± 0.30	4^b^ ± 0.60	5^a^ ± 0.52	8^b^ ± 0.47	6^a^ ± 0.39	8^a^ ± 0.41	10^b^ ± 0.85	8^a^ ± 0.81
	**Overall significance level**												
	Location	Sowing date	Cultivar	Year	Location x Cultivar	Sowing date x cultivar	Cultivar x year						
	***	***	***	***	**	NS	***						

ES, early sowing; CS, conventional sowing; NT, not tested; NS, not significant; means followed by the same letter are not significantly different within Locations, cultivars, year or sowing dates at p < 0.05; *** p < 0.001; **p < 0.01.

Values are an average of 10 simulations (1000 seeds/simulation/cultivar for each sowing date/location/year (360 000 seeds in total).

Seedling mortality due to a soil surface crust ranged from 0 (in En Crambade, except for the early sowing in 2014 and in Mondonville, except for the early sowing in 2013 and conventional sowing in 2014) to 35% (for early sowing in Mondonville, in 2013). There was statistically significant effect of locations (*p*<0.001), sowing dates (*p*<0.001), soybean cultivars (*p*<0.001), and years (*p*<0.001), on seedling mortality due to a soil surface crust. We also found statistically significant location x cultivar (*p*<0.01) and cultivar x year (*p*<0.001) interaction effects on seedling mortality due to a soil surface crust although no sowing date x crop cultivar effect (*p*> 0.05) was observed.

Seedling mortality due to soil aggregates ranged from 10 to 14%. There was statistically significant effect of location (*p*<0.001) on seedling mortality due to soil aggregates. However, no significant effects (*p*>0.05) of sowing dates, cultivars, years or their interactions were found on seedling mortality due to soil aggregates.

The lack of emergence due to non-germination ranged from 1 (for cv. Isidor) to 3% (for cvs. Ecudor and Santana). Indeed, significant effects of crop cultivar (*p*<0.001) was observed on non-germination rate while no significant effects (*p*>0.05) of locations, sowing dates, years or their interactions were observed on non-germination rates.

## Discussion

### Germination Rates

Our study provides a range of reference values for germination of three soybean cultivars from contrasted maturity groups and allows their comparison with information already available in the literature. Indeed, previous studies reported that these parameter values do not generally differ among cultivars, but rather among species (Constantin et al., 2015; Dürr et al., 2016; Tribouillois et al., 2016). Indeed, the base temperatures for germination of the cultivars used in this study were 4, 3, and 3.5°C, for cvs. Ecudor, Isidor, and Santana, respectively, which is very similar to the one reported for soybean (Covell et al., 1986) and cv. ES Pallador belonging to MG I as cv. Isidor (i.e. 4°C; Lamichhane et al., 2020a). Likewise, using these base temperatures, thermal time to reach mid-germination was 18°Cd for all three cultivars, that is almost the same as for cv. ES Pallador as reported previously (i.e. 17°Cd; Lamichhane et al., 2020a). The optimum temperature for germination was 28°C, 30°C, and 30°C for cv. Ecudor, Isidor, and Santana, respectively, which is also similar as that of cv. ES Pallador (i.e. 30°C; Lamichhane et al., 2020a) confirming little difference existing among soybean cultivars in terms of germination parameters. The average base water potential was similar among the three genotypes with corresponding values of −0.58, −0.56, and −0.71 MPa for cultivar Ecudor, Isidor, and Santana, respectively. These values are close to that of cultivar ES Pallador (i.e. −0.67 MPa; Lamichhane et al., 2020a) confirming only little difference existing among genotypes belonging to the same species.

### Emergence Rates and Time

The final emergence rates of the three cultivars were different with a few exceptions (e.g. the early sowing in Mondonville in 2013, both sowings in Rivières in 2013, conventional sowing in Mondonville in 2014). However, final emergence ranking among cultivars was inconsistent between years, which was probably due to uncontrolled factors, such as the different quality of seed lots used between years. The use of the same seed lot in both years would have provided consistent results, but it was not possible given that soybean seeds quality during storage rapidly deteriorates over time with drastic reduction in germination potential (Shelar et al., 2008). Previous studies reported that seedbed sowing conditions have much greater effects than cultivars (i.e. seed and seedling characteristics) on final emergence rates, especially when these conditions are unfavorable for seed germination and seedling emergence (Brunel-Muguet et al., 2011; Constantin et al., 2015; Dürr et al., 2016). We found important variation in terms of soil and weather conditions of the seedbed not only between years, but also among locations and sowing dates that certainly affected seedling emergence rates. This was further confirmed by results of the simulation demonstrating that final emergence rates were affected not only by cultivars but also by location, sowing date, year, and their interactions.

We found only little differences in terms of observed final emergence rates of the three cultivars between the sowing dates. Contrary to our expectation, final emergence rates with the early sowing were even higher in some cases (e.g. in Mondonville in 2013 sowings and in Rivières for both year sowings) compared to the conventional sowing. When final emergence rates were compared between locations, the lowest final emergence rate was observed with conventional sowing (45% in Mondonville, in 2014) and not with early sowing. This is probably because, despite a significant difference in terms of seedbed weather conditions between sowing dates, no severe water stress, nor stress due to low temperatures, were observed during field experiments.

The quality of soybean establishment in the field experiments, expressed as the frequency of poor, good, and very good emergence classes, showed good to very good emergence frequency of soybean in almost all cases. In addition, time to reach the maximum emergence among locations or years was similar. This was mainly due to similar seedbed conditions and interactions across locations. These results are encouraging for farmers explaining that early sowing is unlikely to hinder soybean establishment in Southwestern France, especially when seedbed moisture is not a limiting factor. Therefore early sowing may represent an important agronomic lever to escape summer drought that markedly limit soybean yield in this region.

The number of field counting of emergence courses and final rates were heterogeneous across our study sites. Because we compared three different cultivars across three locations, quite far from each other, we did have very short periods of time available for emergence counting, as often reported (e.g. Dürr et al., 2016). However, the consideration of different crop cultivars, sowing dates, and study locations allowed us to collect a set of seedbed and weather variables to be used in simulation studies with the SIMPLE model. This makes it possible to obtain more reliable statistics concerning the major causes of non-emergence across our regions, and also to compare key factors tested across the study locations *via* simulation studies. This is one of the key benefits of combining modeling to an experimental approach because these comparisons would be untractable *via* field experiments due to the huge work load required, and the associated costs. Indeed, the comparison of the predicted and observed emergence courses, including final rates showed a satisfactory fitting quality of the SIMPLE model, emphasizing the robustness of its prediction quality as reported previously (Constantin et al., 2015; Dürr et al., 2016; Lamichhane et al., 2020a). These results are consistent with the field reality where seedling mortality due to a soil surface crust is much higher in silty soil compared with other soil types.

### Causes of Non-Emergence

A detailed diagnosis of the causes of non-emergence under field conditions is time consuming because one should look for the non-germinated seeds, or non-emerged seedlings under the soil profile. There are two major challenges while making this diagnosis: i) if no timely intervention is made for this diagnosis, due to difficulty in field access or other reasons, non-germinated seeds or non-emerged seedlings are extremely difficult to retrieve (either they are rotten or eaten by predators); ii) once non-germinated seeds or non-emerged seedlings are retrieved, it may be very difficult to identify the precise factors associated with the non-emergence, without expert observations, as a range of biotic and abiotic factors affect the crop establishment phase (Lamichhane et al., 2018). More specifically to our case, this difficulty was further exacerbated by the fact that our experiments were conducted across different locations and at different sowing dates, using three different cultivars. Therefore, we performed simulation studies that helped identify major causes of non-emergence, as the SIMPLE model finely predicts these causes (Dürr et al., 2016; Lamichhane et al., 2019; Lamichhane et al., 2020a).

Simulation results showed that non-germination rates were significantly affected by cultivars (thus seeds and seedling characteristics) and not by seedbed conditions. This ranking in terms of non-germination rates of the three cultivars was clearly affected by the parameter values calculated by the laboratory experiments. Field observations would have provided better insights concerning the potential role of seedbed weather conditions on non-germination rates. However, this was not possible due to very frequent rainfalls that limited filed access as well as the intense time and resource needed to make these measurements at the same time across different sites. The average quantity of cumulated rainfall 30 days after sowing ranged between 34 and 125 mm across our study sites. This amount of rainfall is higher than the average amount of rainfall that generally occurs in Southwestern France. This also suggests that no severe drought stress was occurred in the seedbed during the study periods that otherwise would have affected seed germination rates.

The impact of seedling mortality rates due to a soil surface crust was the most variable among all causes of non-emergence ranging from null to very high (35%; **Table 6**). These rates were directly dependent on the soil type and the quantity of cumulated rainfall after sowing. For example, seedbed in Mondonville for all sowings and in Rivières in 2013 sowings was characterized by loamy to very loamy soil that are very sensitive to soil surface crusting in the presence of rainfall. At the same time, the frequency of rainfall events 30 days after sowing was very high across these locations favoring the formation of a soil surface crust that hindered seedling emergence. In contrast, seedling mortality due to soil surface crusting was much lower for clay soil seedbeds that are more resistant to soil crust. These results corroborate previous studies that reported the effect of soil texture and seedbed structure on the frequency of soil surface crusting in relation to the cumulated rainfall (Govers et al., 1990; Gallardo-Carrera et al., 2007). Results of our simulation study further confirmed a significant effect of sowing conditions (location, sowing date, year, and their interactions) on seedling mortality rates due to a soil surface crust.

Simulated seedling mortality rates due to soil aggregates were very similar with little variability among sowing dates, years, cultivars or their interactions highlighting no potential effect of these factors. This was because we chose a coarse seedbed structure, resulting from conventional tillage that is very common in Southwestern France (Lamichhane et al., 2020a).

We did not focus on the potential effect of biotic stresses on soybean establishment in field or simulation studies. This is the main limit of this study. Nevertheless, we covered our experimental plots with nylon nets above soil surface, since seeds and seedlings damages due to vertebrate pests, including birds and wild rabbits are frequently observed across South-West France. In contrast, no important emergence losses due to other biotic stresses have been reported to date in France (Lamichhane et al., 2020a). Biotic stresses, such as soil-borne diseases, however could be a limiting factor for a good quality of soybean establishment. This will be emphasized if this crop is more frequently grown under early sowings in the future.

### No Correlation Between Average Emergence Rate and Grain Yield

We did not find any positive correlation between the seedling emergence rate and grain yield of soybean (**Table 3**). This is not surprising for three key reasons. First, the sowing density used was much higher than the limiting densities for grain yield, to compensate any potential seedling emergence losses. Indeed, yield plateaus are reported to occur at densities of 23.0 and 19.9 plants m^−2^ for new and old soybean cultivars, respectively (De Bruin and Pedersen, 2009). Second, unlike crops such as sunflower or maize, soybean has a high degree of plasticity in vegetative traits and thus is capable of compensating seedling emergence losses due to its semi-determinate or indeterminate growth habit and ramification capacity (Vega and Sadras, 2003; Andrade and Abbate, 2005). Indeed, the cultivar Isidor used in this study is characterized by semi-determinate growth while cultivars Ecudor and Santana are characterized by indeterminate growth (Schoving et al., 2020). However, the rate of soybean emergence may significantly impact grain yield when a lower density than used herein is adopted and when cultivars with determinate growth habit is used (Vega and Sadras, 2003). Third, because many biotic and abiotic factors affect a given crop during its growth cycle, any crop experiencing such stress factors post-emergence may have a significant impact on final yield. For example, soybean in southern Europe is subjected to severe drought stress during its flowering phase that severely impact grain yield (Maury et al., 2015). Therefore, it is evident that, in such a case, a high emergence rate does not ensure a high grain yield.

### The Use of Crop Emergence Models Provides Complementary Data to Field Experiments

Field experiments on seed germination and emergence dynamics are often difficult to realize because this phase, especially when seedbed conditions are favorable for the crop, occurs very rapidly. In addition, favorable seedbed conditions for the crop could also mean unfavorable ones for field access and measurements (e.g., frequent rainfall events that limit field access; Lamichhane et al., 2019). In such situations, a delay of a few days could jeopardize measurements of seed germination and seedling emergence dynamics. This is especially true when one considers a wide range of factors (different sowing dates, locations, and cultivars). Simulation studies using a crop emergence model represent a powerful means to overcome this limit. In particular, the use of a crop emergence model, such as SIMPLE, can markedly facilitate a better understanding of emergence courses, final rates and causes of non-emergence, as the quality of prediction of this model is robust enough, as demonstrated for an number of crops (Moreau-Valancogne et al., 2008; Constantin et al., 2015; Dürr et al., 2016; Lamichhane et al., 2020a). This was further confirmed by the comparison of observed and simulated results of this study.

## Conclusions

This study evaluated the quality of soybean emergence as affected by different cropping practices (cultivars from different maturity groups and different sowing dates) and a wide range of sowing conditions (locations and years). This allowed us to evaluate the potential effects of cultivar, environment, cropping practices, and their interactions on final emergence rates of soybean. Our main finding is that early sowing is unlikely to affect soybean emergence and stand development in South-West of France and therefore may represent an important agronomic lever to escape summer drought that markedly limit soybean yield in this region. This is also because field access, unlike in North European conditions, is not a limiting factor in this region, due to a lower frequency of rainfall. Although we used cultivars belonging to three MG (I, II, and I/II), it would be interesting in the future to increase the range of genotypes, especially those belonging to earliest maturity group (i.e. MG 000) that are especially adapted to Northern European growing conditions.

The results of model simulations suggest that seedling mortality under a soil surface crust, especially in silty soil, and that due to soil aggregates are two key factors reducing soybean emergence. Both of these mechanical stresses result as a consequence of practicing tillage and that almost 95% of French growers perform tillage prior to sowing soybean. This suggests that, growing soybean under no-till conditions could improve final emergence rates of this crop. At the same time, direct sowing may provide higher possibility for growers to perform early sowing as frequent rainfall during early spring often limits field access for growers in conventional system. This is especially true under North European conditions, where soybean acreage is expected to increase in the next decades. Early sowing under no-till conditions could thus be particularly interesting for soybean growers to reduce production costs related to tillage practices. However, biotic stresses, especially seed predation due to vertebrate and invertebrate pests, could cause emergence losses under no-tilled conditions which needs further investigation.

## Data Availability Statement

The datasets presented in this study can be found in online repositories. The names of the repository/repositories and accession number(s) can be found in the article/**Supplementary Material**.

## Author Contributions

LC, PD, and PM, designed the research. LC, PD, and PM acquired the funding for the research project. LS, PD, and PM performed the experiments. JL analyzed the data. JL, J-NA, and PM wrote the manuscript.

## Conflict of Interest

The authors declare that the research was conducted in the absence of any commercial or financial relationships that could be construed as a potential conflict of interest.

## Supplementary Material

Supplementary Material S1 Raw data on the response of the three cultivars to the tested temperatures and water potentials.

Supplementary Material S2 Weather data of the three study sites over 2013–2014.

Supplementary Material S3 Number of days from emergence to maturity of the three soybean cultivars grown over space and time under two irrigation regimes.
